# Clinical Significance and Resource Burden of Double Duct Sign in Non-jaundiced Patients

**DOI:** 10.7759/cureus.56252

**Published:** 2024-03-16

**Authors:** Ahmed Mahmoud Askar, Wajith Hussain Zahir Hussain, Rishi Chavda, Wen Chung, Eyad Issa, John Isherwood, Ashley Dennison, Giuseppe Garcea

**Affiliations:** 1 Department of Hepatopancreatobiliary (HPB) Surgery, University Hospitals of Leicester NHS Trust, Leicester, GBR

**Keywords:** dilated pancreatic duct, ampullary tumour, pancreatic malignancy, dilated common bile duct, double duct sign

## Abstract

Aim

The study aims to determine the incidence of malignancy at presentation and subsequent risk of malignancy (at 12 months follow-up) in a cohort of patients with double duct sign (DDS) on cross-sectional imaging but no visible stigmata of jaundice. The study also correlates malignancy with liver enzyme dysfunction and estimates the resource burden incurred during the investigation of these patients.

Methods

A search for the key term "double duct sign" was undertaken in the radiological database of a tertiary hepatopancreatobiliary (HPB) centre between March 2017 and March 2022. Radiological reports, clinic letters, blood results, and multidisciplinary team meeting (MDT) outcomes were reviewed during this period and at one year. The national tariff payment system was reviewed to identify tariffs for different investigations required for the cohort and to calculate the total cost incurred.

Results

Ninety-seven patients with DDS were identified. Sixty-four patients (66%) had a normal bilirubin (0-21 µmol/L) at presentation and were included in the analysis. Seven patients (10.9%) were diagnosed with malignant peri-ampullary tumours, and 21 (32.8%) were diagnosed with benign diseases. In 34 patients (53%) with DDS, the underlying cause remained uncharacterised. Most patients had mild abnormalities of liver enzymes, but two patients (4.3%) were diagnosed with malignant peri-ampullary tumours despite having normal serological values. Patients who had a benign diagnosis and/or who had cancer excluded without a definitive diagnosis did not go on to develop a malignancy at 12 months follow-up. However, in those patients where the underlying aetiology could not be characterised, extended surveillance was required with a total of 80 MDT discussions and multiple surveillance scans (103 CT and 65 MRI scans). Twenty-six patients underwent endoscopic ultrasound (EUS) with three patients requiring more than one EUS examination (29 investigations in total). The cost of these investigations was £38,926.89.

Conclusion

This study confirms that DDS even in patients without clinical jaundice or with normal liver enzymes requires careful investigation to exclude malignancy despite the resource burden this entails. This supports previously reported results in the literature, and despite the increased use of cross-sectional imaging, DDS remains a clinically significant finding. Large cohort risk stratification studies would be useful to determine clinical urgency and allow the appropriate allocation of resources.

## Introduction

Double duct sign (DDS) is the simultaneous dilatation of the common bile duct (CBD) and the pancreatic duct (PD) [1], first described by Freeny et al. in 1976 in a series of endoscopic retrograde cholangiopancreatography (ERCP) [2]. It is always assumed that DDS is a sign of malignancy in the peri-ampullary region especially if associated with overt jaundice, a high bilirubin level, or deranged liver function tests (LFTs) [3]. Nevertheless, the incidence of tumours in DDS incidentally found when associated with normal LFTs remains unclear, and the incidence is increasing due to the almost ubiquitous use of cross-sectional imaging. Benign causes for DDS were also reported including CBD stones [4], chronic pancreatitis [5], and sphincter of Oddi dysfunction [6,7]. DDS often results in multiple discussions at multidisciplinary team meetings (MDT) and further investigations including endoscopic ultrasound (EUS) and/or surveillance scans.

In this paper, we examine a cohort of patients with DDS on cross-sectional imaging (often as incidental finding) but with normal bilirubin levels. The malignancy rate at presentation and after 12 months of surveillance is reported together with the overall resource burden incurred.

This article was previously presented as a conference abstract at the Pancreatic Society of Great Britain and Ireland (PSGBI) 2023 48th Annual Meeting on November 30, 2023.

## Materials and methods

The radiology department in our hepatopancreatobiliary (HPB) tertiary centre was asked to conduct a search for patients where a DDS was reported following their cross-sectional imaging (i.e., computed tomography (CT) and magnetic resonance imaging (MRI)) over the preceding five years. Multiple keywords were used to ascertain capturing the right cohort including double duct sign, dilated pancreatic duct, and dilated common bile duct. Ninety-seven patients were identified in the period between March 2017 and March 2022. Imaging reports were reviewed, and 64 (66%) were found to have at least one cross-sectional imaging modality showing a DDS with a normal bilirubin level (0-21 µmol/L). Thirty-three patients were excluded due to jaundice at presentation (bilirubin >21 µmol/L). We also examined a subgroup of 46 patients (47.4%) who had completely normal LFTs in addition to a normal bilirubin (0-21 µmol/L), alkaline phosphatase (ALP; 30-130 IU/L), and alanine transaminase (ALT; 2-53 U/L) to determine whether the risk of malignancy was different if all LFTs were considered and not simply bilirubin in isolation. Tumour markers were reviewed when available, specifically cancer antigen 19-9 (CA19-9) and carcinoembryonic antigen (CEA).

The cohort's radiological reports, clinic letters, blood results, endoscopy reports, and MDT outcomes were reviewed during the period of presentation and at 12 months follow-up to ensure that delayed presentation of malignancy was captured. We also reviewed the number of additional investigations and MDT discussions generated as a result of the finding of a DDS during the first 12 months after presentation; the national tariff payment system was reviewed to identify tariffs for CT and MRI scans and EUS including reporting. Tariffs of additional investigations were added to calculate the subsequent cost and resource implications on the trust.

## Results

**Figure 1:**
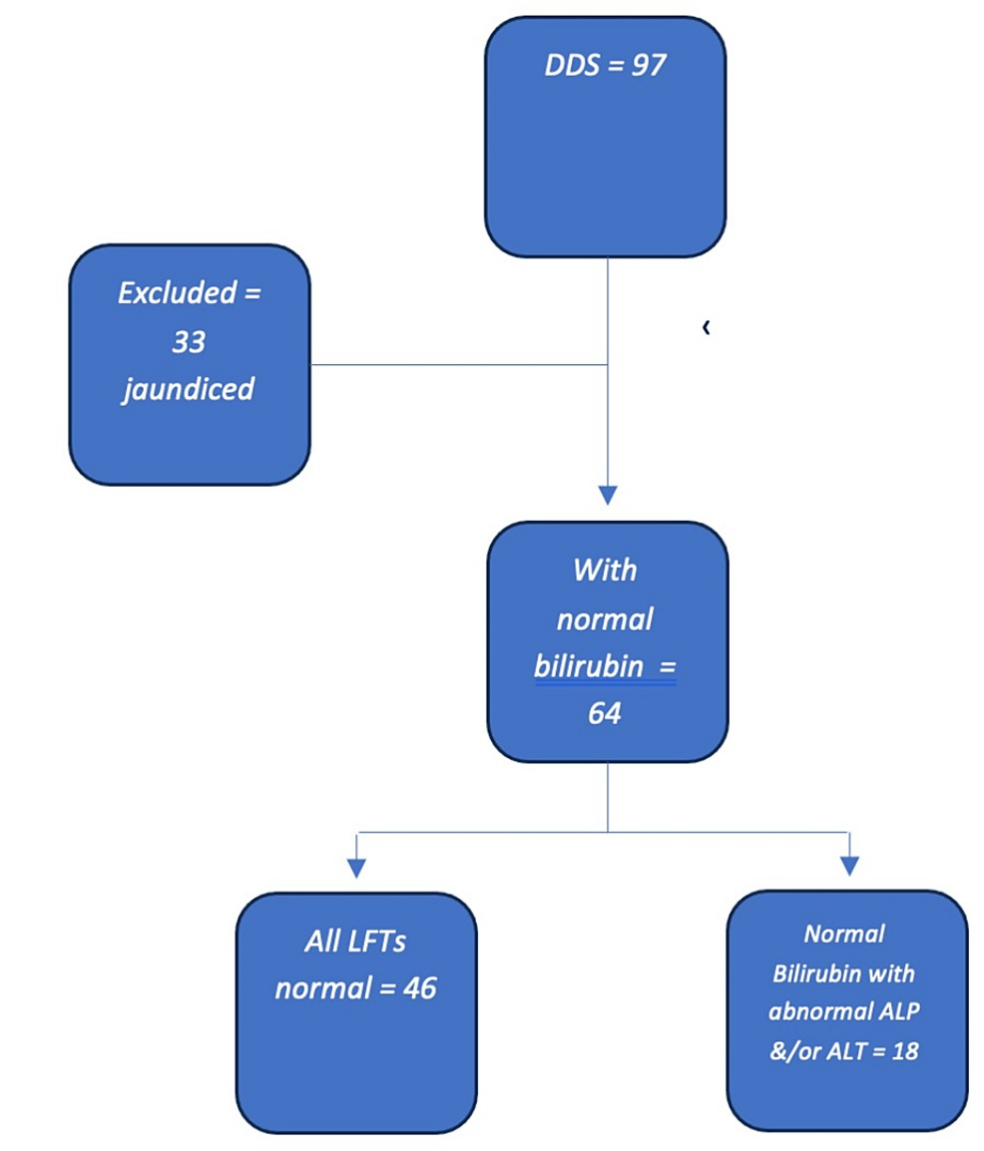
Patients' inclusion criteria DDS: double duct sign; LFTs: liver function tests; ALP: alkaline phosphatase; ALT: alanine transaminase

Ninety-seven patients who had cross-sectional imaging modalities reporting a DDS in the period between March 2017 and March 2022 were identified, of whom 64 were not clinically jaundiced (no visible jaundice and with normal bilirubin level regardless of other LFT results) (Figure 1). In the excluded 33 patients who were jaundiced at presentation, the incidence of malignancy was high with 23 (69.9%) having a confirmed diagnosis. The age range of this group of patients was 33-93 years with a median of 76 years. For the serological tests, the median values were as follows: bilirubin 154 µmol/L, ALP 578 IU/L, ALT 262 U/L, CA19-9 267 U/ml, and CEA 2.5 U/ml.

In the included group of 64 patients (23 males and 41 females), the age range was 43-97 years with a median of 75.5 years. The median serological tests were as follows: bilirubin 10 µmol/L, ALP 95 IU/L, ALT 25 U/L, CA19-9 16 U/ml, and CEA 2 U/ml. Seven patients (10.9%) were diagnosed with malignant peri-ampullary tumours, 21 (32.8%) were diagnosed with benign disease, and two (3%) had indeterminate lesions, but in 34 patients (53%), the aetiology remained uncharacterised (Table 1).

**Table 1:** Comparison of criteria and results of all groups ALP: alkaline phosphatase; ALT: alanine transaminase; CA19-9: cancer antigen 19-9; CEA: carcinoembryonic antigen; LFTs: liver function tests

Group	Number	Median age (years)	Median bilirubin (µmol/L)	Median ALP (IU/L)	Median ALT (U/L)	Median CA19-9 (U/ml)	Median CEA (U/ml)	Malignancy	Benign	Unable to characterise
Jaundiced	33	76	154	578	262	267	2.5	23 (69.9%)	7 (21.2%)	3 (9%)
Non-jaundiced: normal bilirubin	64	75.5	10	95	25	16	2	7 (10.9%)	21 (32.8%)	34 (53%)
Normal LFTs	46	76.5	9.5	81.5	23	16	2	2 (4.3%)	14 (32.8%)	28 (60.8%)
Non-jaundiced with malignancy	7	75	11	197	204	226	3	7	-	-

The most common imaging modality was CT scan (CT abdomen and pelvis, CT colonography, and CT angiography) followed by US, MRI, and bone scans (Table 2). Indications for the imaging modalities included surveillance for a different condition including previous colorectal cancer, non-specific abdominal pain, weight loss, shortness of breath, dyspepsia, iron deficiency anaemia, falls, and back pain (Table 3). The smallest CBD diameter reported where the description included the term double duct sign was 10 mm (range 10-27 mm, median 12 mm), and the smallest PD diameter was 3.4 mm (range 3.4-36 mm, median 6 mm). Malignant lesions included tumours of the head of the pancreas in four patients, cholangiocarcinoma in two, and a peri-ampullary tumour in one. In those patients where a benign cause was found, the most common aetiology was chronic pancreatitis in nine patients followed by CBD stones in four, benign tumours in three (two side-branch intraductal papillary mucinous neoplasms (SB-IPMN) and one adenoma), blocked CBD stents in two, benign strictures in two, and benign CBD polyp in one.

**Table 2:** First imaging modality identifying DDS DDS: double duct sign; CT: computed tomography; US: ultrasonography; MRI: magnetic resonance imaging

Imaging modality	Number
CT abdomen and pelvis	31
US abdomen and pelvis	17
CT angiography	6
MRI abdomen	6
CT colon	3
Bone scan	1

**Table 3:** Presenting complaint

Presenting complaint	Number
Surveillance for other conditions	9
Weight loss	8
Abdominal pain	8
Short of breath	5
Dyspepsia	4
Change in bowel habits	3
Iron deficiency anaemia	2
Feeling abdominal mass	2
Itchiness	1
Fall	1
Claudication	1
Back pain	1

Fifty-five of these patients were discussed in the MDT due to clinical uncertainty where the initial cross-sectional imaging modality failed to confidently identify the cause of the DDS. Many of these patients were discussed on multiple occasions, and the total number of MDT discussions in this group was 80. Multiple scans at presentation and at intervals were also requested; the national tariff payment system was used to assess the individual cost of the investigations [8]. A total of 103 CT scans cost the trust £11,021 (tariff £107 including reporting), and a total of 65 MRI scans cost £8580 (tariff £132 including reporting). Twenty-six patients had an EUS with some patients requiring more than one EUS (total of 29) costing the trust £19,325.89 (code GB13Z=£666.41). This cohort consequently cost the trust a total of £38,926.89 despite being unable to confirm a sinister pathology or reach a definitive diagnosis in the majority of patients.

We also reviewed a subgroup of 46 patients who had completely normal LFTs in addition to having normal bilirubin. There were 18 males and 28 females; the age range was 43-97 years and the median age was 76.5 years with median serological values as follows: bilirubin 9.5 µmol/L, ALP 81.5 IU/L, and ALT 23 U/L. In this subgroup, only two patients (4.3%) had a diagnosis of a malignant peri-ampullary tumour (one cholangiocarcinoma and one head of the pancreas tumour). Fourteen patients (32.8%) had benign causes, and the majority had a malignant aetiology excluded but no definitive diagnosis could be identified (28 patients (60.8%)) (Table 1).

Malignancy rate in incidental DDS in non-jaundiced patients

In seven patients, there was a diagnosis of malignancy (three males and four females, age range 63-93 years old, median 75 years). Two patients had completely normal LFTs with the remainder having normal bilirubin levels but markedly deranged ALP (>130 IU/L) and/or ALT (>53 U/L) with median serological values as follows: bilirubin 11 µmol/L, ALP 197 IU/L, and ALT 204 U/L. Weight loss was noted in four patients, and the CA19-9 level was elevated in two (430 and 5617 U/ml) (Table 4).

**Table 4:** Demographics and results of the non-jaundiced who developed malignancy ALP: alkaline phosphatase; ALT: alanine transaminase

Age	Gender	Presenting complaint	Bilirubin (µmol/L)	ALP (IU/L)	ALT (U/L)	Diagnosis
96	Female	Weight loss	Normal	Normal	Normal	Cholangiocarcinoma
71	Male	Abdominal pain	Normal	Normal	Normal	Head of the pancreas tumour
93	Female	Abdominal pain	Normal	870	121	Head of the pancreas tumour
86	Female	Abdominal pain	Normal	197	204	Head of the pancreas tumour
75	Female	Weight loss	Normal	761	315	Peri-ampullary tumour
64	Male	Weight loss	Normal	-	228	Cholangiocarcinoma
63	Male	Weight loss	Normal	538	388	Head of the pancreas tumour

Follow-up at 12 months

None of the patients diagnosed with a benign disease or where a malignant cause was excluded but no definitive diagnosis was reached developed any evidence of tumour development at one-year follow-up.

## Discussion

Our results have confirmed that despite the increased use of cross-sectional imaging, the significance of DDS, particularly when associated with clinical jaundice, is associated with underlying malignancy in 69.6% of the patients. Krishna et al. reported a peri-ampullary malignancy incidence of 85.5% amongst a cohort of 166 patients presenting with DDS associated with jaundice [9]. In the absence of clinical jaundice, the incidence of malignancy is considerably reduced but remains significant at 10.9%. Even in those patients with completely normal liver enzymes, 4.3% will have an underlying malignant diagnosis which given the impetus for early surgery on patients with peri-ampullary malignancies remains a very important consideration. Of interest, in the majority of patients, no definitive diagnosis could be made to explain the DDS although malignancy was confidently excluded following further investigations and was evident at 12 months follow-up (53%). Yao et al. have followed up a similar cohort for five years, and none of the 21 patients with incidental DDS with normal LFTs were diagnosed with peri-ampullary malignancy after five years [10].

Risk stratification and role of EUS

It is difficult to identify specific risk factors for malignancy in those presenting with DDS with no clinical jaundice or with normal LFTs due to the small number of patients in this study. The cohort also represented a markedly heterogeneous group with considerable variation in age, gender, and presenting complaints. The advent of EUS enables detailed imaging of the peri-ampullary area, and it is increasingly used to confidently confirm or refute malignancy. A systematic review by Kanchustambam et al. assessed 177 patients in four studies where an imaging modality had demonstrated a DDS with uncharacterised pathology identified. In this group, the EUS detection rate for peri-ampullary tumours was 5% (CI 95%, 0-10%), and there was a benign pathology detection rate of 22% (CI 95%, 10-34 %). Sixty-nine percent of the patients had normal EUS findings (95% CI, 55-84%) despite DDS being reported in the cross-sectional imaging; therefore, EUS was recommended for these patients to overcome the high false-negative detection rate found with other imaging modalities (in these studies prior to the advent of EUS) [11]. Cohen et al. reviewed 68 non-jaundiced patients who underwent EUS evaluation of DDS without mass detected on cross-sectional imaging; 91% of the patients had a benign cause, while 9% had ampullary malignancy detected [12]. This has encouraged most centres to use EUS as a promising tool in the assessment of these patients [13]. Although other more recent studies have examined cross-sectional imaging modalities and discussed approaches to increase the sensitivity in patients with DDS, EUS remains at present the gold standard modality [14,15].

Limitations

Data extraction was difficult due to the retrospective nature of the study, and different keywords were used to make sure all the patients were captured (double duct sign, dilated pancreatic duct, and dilated common bile duct). Another limitation of this study was the small number of patients diagnosed with peri-ampullary malignancy, which didn't allow enough data for risk stratification as mentioned above.

## Conclusions

This study confirms that DDS even in patients without clinical jaundice or with normal liver enzymes requires careful investigation to exclude malignancy despite the resource burden this entails. This supports previously reported results in the literature, and despite the increased use of cross-sectional imaging, DDS remains a clinically significant finding. Large cohort risk stratification studies would be useful to determine clinical urgency and allow the appropriate allocation of resources.
